# Significance of EGFR and PTEN Expression and PLR and NLR for Predicting the Prognosis of Epithelioid Malignant Peritoneal Mesothelioma

**DOI:** 10.1155/2019/7103915

**Published:** 2019-02-04

**Authors:** Yufei Liang, Guoqi Zheng, Wenjie Yin, Hui Song, Chunying Li, Liang Tian, Dongliang Yang

**Affiliations:** ^1^Department of Gastroenterology, Cangzhou Central Hospital, Cangzhou, Hebei 061001, China; ^2^Department of Pathology, Cangzhou Central Hospital, Cangzhou, Hebei 061001, China; ^3^Cangzhou Medical College, Cangzhou, Hebei 061001, China

## Abstract

**Objective:**

The aim of our study was to investigate the expression of EGFR and PTEN in tissues and measure the serum platelet-to-lymphocyte ratio (PLR) and neutrophil-to-lymphocyte ratio (NLR) to evaluate the prognostic factors of patients with epithelioid malignant peritoneal mesothelioma (MPeM).

**Methods:**

33 patients of pathologically diagnosed epithelioid MPeM tissues were analyzed using immunohistochemistry to detect EGFR and PTEN; the PLR and NLR were determined by using a routine blood test. We analyzed the relationships of these markers to age, sex, asbestos exposure, elevated platelet count, ascites, and clinical stage.

**Results:**

EGFR and PTEN expressions were positive in 22 (66.67%) and 7 (21.21%) epithelioid MPeM patients, respectively. However, these two markers as well as PLR and NLR were not significantly associated with age, sex, asbestos exposure, elevated platelet counts, ascites, and clinical stage (*P* > 0.05). The correlation between EGFR and PTEN was negative (*r* = −0.577, *P* < 0.001), but the correlation between NLR and PLR was positive (*r* = 0.456, *P* = 0.008). The median survival of all patients was 6 months. In univariate analysis, PTEN (*P* < 0.001), PLR (*P* = 0.014), and NLR (*P* = 0.015) affected the overall survival. Multivariate analysis revealed that PTEN and PLR were validated as predictive for overall survival of epithelioid MPeM (HR = 0.070, *P* = 0.001, and HR = 3.379, *P* = 0.007, respectively).

**Conclusion:**

On the basis of these results, it is suggested that PTEN and PLR are risk factors for the prognosis of epithelioid MPeM, which may be targets for selective therapies and improve the outcomes of patients with epithelioid MPeM.

## 1. Introduction

Malignant peritoneal mesothelioma (MPeM) is a rare neoplasm arising from the serosal lining of the peritoneal cavity and is related to asbestos exposure in most cases [1]. Like pleural mesothelioma, it is quite aggressive, with most patients succumbing to this disease within 7-14 months after diagnosis [2]. The histology of MPeM is divided into epithelial type, sarcoma type, and mixed type, among which epithelial type accounts for the majority [1]. Although diagnostic techniques and treatment of MPeM have improved, prognosis is poor. Therefore, it is critically important to identify factors to predict prognosis to develop treatments.

Researchers agree that the tumor microinflammatory state of the body and the body's immune system can significantly affect prognosis [3], and variations in systemic inflammatory response biomarker levels have been associated with adverse clinical outcome in various malignancies. Inflammation leading to oxidative stress and cell damage contributes to causing genetic alterations that trigger malignant transformation, and the ensuing inflammatory response continues to fuel tumor progression. The platelet-to-lymphocyte ratio (PLR) and neutrophil-to-lymphocyte ratio (NLR) serve as markers of inflammation and prognosis of patients with solid tumors that can be detected early [4–6], but in several reports, NLR exhibited no predicted power on overall survival in malignant mesothelioma [7, 8].

Tumor invasion and metastasis are complex, multistep processes driven by oncogenes and tumor suppressor genes, abnormal signal transduction pathways, and abnormal cell cycle regulation, which mediate tumorigenesis and disease progression. The phosphatidylinositol 3-kinase (PI3K)/mammalian target of rapamycin (mammalian target of rapamycin (mTOR)) pathway has been the focus of interest in identifying potential prognostic markers of cancer [9]. Activation of the PI3K/mTOR pathway regulates cell growth, protein biosynthesis, and proliferation, which promote tumorigenesis [9].

EGFR is the titular member of a family of receptor tyrosine kinases (RTKs), which transduces signals to intracellular signaling systems [10]. PTEN encodes a tumor suppressor with phosphatase activity [11]. EGFR and PTEN regulate the induction and progression of malignant tumors through the PI3K/mTOR signal transduction pathway [12, 13]. Abnormal EGFR and PTEN signaling pathways may produce malignant tumors.

PTEN is expressed by malignant pleural mesothelioma (MPuM) cells [14], and PLR and NLR are associated with prognosis in cancer patients [15]. In contrast, clinical studies of EGFR expression in MPeM are few and are based on relatively small numbers of tissue patients [16]. However, there has been no previous validation confirming the relationship of all four indicators (including PTEN, EGFR, PLR, and NLR) and survival in epithelioid MPeM. Therefore, our aim was to validate the prognostic role of four indicators for overall survival (OS) in epithelioid MPeM.

## 2. Materials and Methods

Consecutive patients with a diagnosis of epithelioid MPeM made between 1 January 2013 and 31 December 2015 who were diagnosed at or attended in Cangzhou Central Hospital were included in this retrospective study. This study was approved by the human ethics committees of Cangzhou Central Hospital (approval ref. no. 2012-012-01) to acquire paraffin-embedded peritoneal tissues from 33 patients using B-mode ultrasound-guided biopsy or surgery. Patients did not receive antitumor treatment. The pathological diagnosis was confirmed by two experienced pathologists according to the 2012 update of the United States “Mesothelioma Pathology Diagnosis Guide” [17]; the results and quality control standards for each assay were independently reviewed. MPeM is clinically divided into 4 phases as follows: (I) tumor confinement to the peritoneum; (II) tumor invasion to the peritoneal surface of the organ, the abdominal diaphragm, and/or the abdominal lymph nodes; (III) tumor metastasis to the lymph nodes outside the abdominal cavity; and (IV) blood transfer to distant organs [18].

All specimens were fixed using 10% neutral formalin, embedded in paraffin, and subjected to immunohistochemical analysis using the ABC method. The standard streptavidin biotin peroxidase detection technique was used as described [19]. A 4 *μ*m tissue section of each specimen was deparaffinized and rehydrated. The citrate buffer microwave was used for antigen retrieval, and 3% hydrogen peroxide blocked the endogenous enzyme to reduce background staining. After adding the primary antibody at 4 degrees overnight, and washing with PBS, a biotinylated secondary antibody was added to slides, horseradish peroxidase was used as a marker, and diaminobenzidine was used as a chromogen. The negative control was a slide prepared in parallel without the primary antibody.

### 2.1. Blood Samples

Platelet, neutrophil, and lymphocyte numbers were determined using routine blood tests. The PLR was calculated as the ratio of the count of platelet to lymphocyte. The median PLR value was used because the data was not normally distributed. The median PLR value was 278, which was used as cutoff to classify MPeM as ≥278 group versus <278 group. The NLR was derived by dividing the absolute neutrophil count by the absolute lymphocyte count, and a cutoff of <5 vs ≥5 was used in accordance with the first report of NLR in MPeM [20].

### 2.2. Reagent

A rabbit anti-human EGFR monoclonal antibody (clone number: 31G7) and a rabbit anti-human PTEN polyclonal antibody (clone number: 28H6) were purchased from Beijing ZS Biological Technology Co., Ltd. The DAB Reagent (ZLI-9032) was purchased from the Beijing ZS Company.

### 2.3. Interpretation of Immunohistochemical Results

EGFR is based on a membranous staining pattern (brown-yellow or brown granules), and PTEN is detected in the cytoplasm (brown granules). Absence of specific staining of tumor cells was considered negative. Two pathologists, who were not informed of the origins of the tumor tissue samples, evaluated the staining patterns. Immunohistochemical staining results were judged according to a standard protocol that employed optical microscopic observations (×400 magnification) of five to eight randomly selected horizons. The sections were semiquantitatively assessed for the PTEN and EGFR expression by an observer. The intensity of staining and the percentage of positive cells were scored semiquantitatively 0 (negative; <5% of immunoreactive cells), 1 (weak; 5–25% of immunoreactive cells), 2 (moderate; 26–50% of immunoreactive cells), and 3 (strong; >50% of immunoreactive cells) [21]. The score ≥1 was regarded as positive; otherwise, the judgment was negative.

### 2.4. Follow-Up

Patients' survival was defined according to the date of pathological diagnosis of epithelioid MPeM. Patients were followed monthly until 31 December 2016 by telephone calls or an outpatient referral form. None of the patients was lost to the study.

### 2.5. Statistical Analysis

The statistical analyses were performed using SPSS 19.0. Chi-square test and Fisher exact test were used to test the association variables for categorical data. Correlations between parameters were tested by calculation of the Spearman rank correlation coefficient. For each variable, we use the Kaplan–Meier method with two-tailed log-rank *P* values to evaluate potential prognostic factors. Then, we incorporate meaningful factors from univariate analysis into Cox's proportional hazards regression model and forward LR method as multivariate analysis to estimate the independent prognostic predictor(s). *P* < 0.05 was considered statistically significant; however, in the univariate analysis, we set *P* < 0.02 to be statistically significant for inclusion in multivariate analysis.

## 3. Results

### 3.1. Patients' Demographic Data

A total of 33 patients met the inclusion criteria and were included in the analyses. The patients (9 men and 24 women) ranged in age from 42 to 75 years (average 61.15 years). Among them, 29 had a history of asbestos exposure history, 24 had elevated platelet counts, and 27 had ascites. According to the clinical stage system, 21 patients (63.64%) were in stages I + II, and 12 patients (36.36%) were in stages III + IV. 14 (42.42%) patients received systemic or local chemotherapy (platinum-based or pemetrexed-based or combined), whereas the remaining patients received the best supportive care or were not treated. The median PLR and NLR were 278 and 5.97, respectively (Table 1).

### 3.2. Expression and Correlation of EGFR, PTEN, PLR, and NLR with the Clinicopathological Features of Patients with Epithelioid MPeM

**Figure 1:**
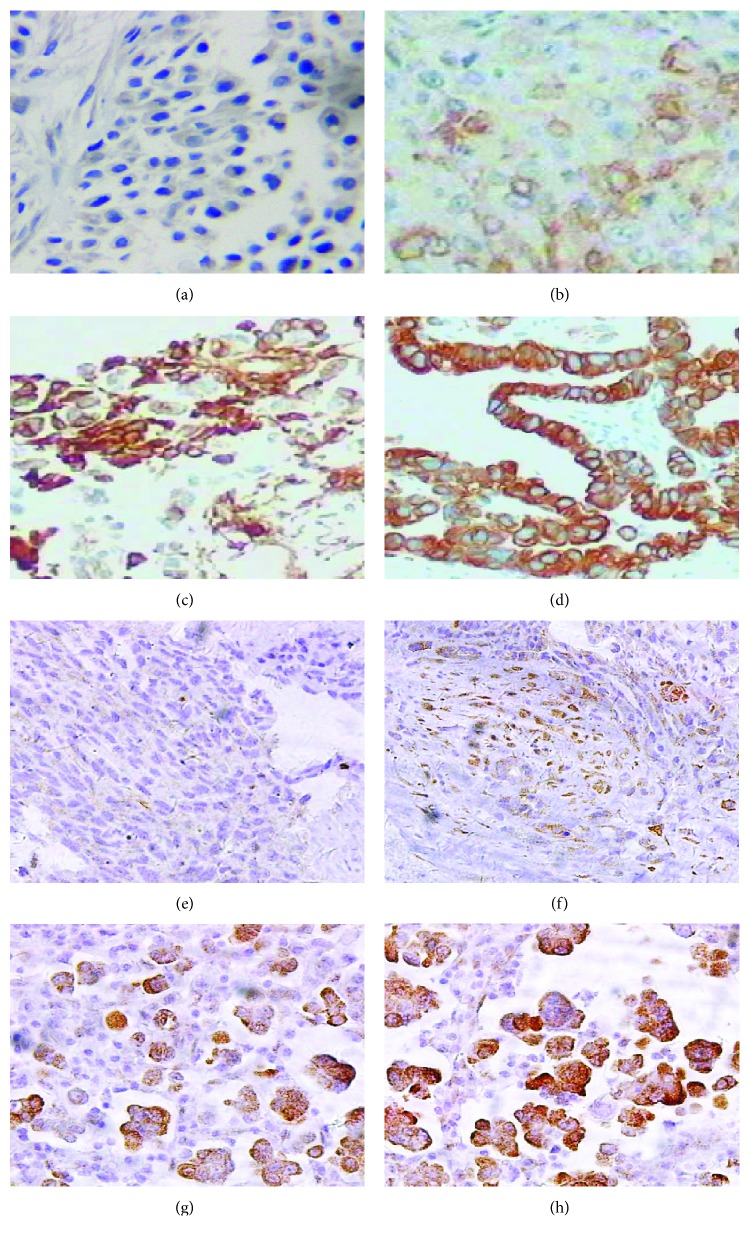
Expression of EGFR and PTEN in epithelioid MPeM tissue. EGFR was based on a membranous staining pattern, and PTEN was detected in the cytoplasm (a and e). Negative for EGFR and PTEN expression. (b and f) Weak expression of EGFR and PTEN in epithelioid MPeM tissue; minority of peritoneal cells are stained yellow. (c and g) Moderate expression of EGFR and PTEN in epithelioid MPeM tissue; a medium quantity of peritoneal cells are stained yellow or brown. (d and h) Strong expression of EGFR and PTEN in epithelioid MPeM tissue; majority of peritoneal cells are stained yellow or brown (×400).

EGFR is mainly stained in the cell membrane, while PTEN is mainly stained in the cytoplasm. Carcinomas expressed EGFR in 22 (66.67%) of 33 specimens and PTEN in 7 (21.21%) specimens (Figure 1). The data for the four indicators were not significantly associated with age, sex, asbestos exposure, elevated platelet count, ascites, and clinical stage (*P* > 0.05). Spearman's rho analysis revealed that the expression of EGFR was negatively correlated with that of PTEN (*r* = −0.577, *P* < 0.001) (Table 2), and NLR was positively correlated with PLR (*r* = 0.456, *P* = 0.008) (Table 3); however, PLR and NLR were not significantly associated with EGFR or PTEN expression (*P* > 0.05).

### 3.3. Survival Analysis

Data were available for all of 33 epithelioid MPeM patients; the follow-up rate was 100%. By the end of the observation period, 30 patients had died and 3 patients remained alive. The 1- and 2-year overall survival rates were 19.6% and 4.4%, respectively. The median survival of the deceased patients after diagnosis was 6 months. The patients surviving at the end of the study had been followed for a median duration of 14 months.

**Figure 2:**
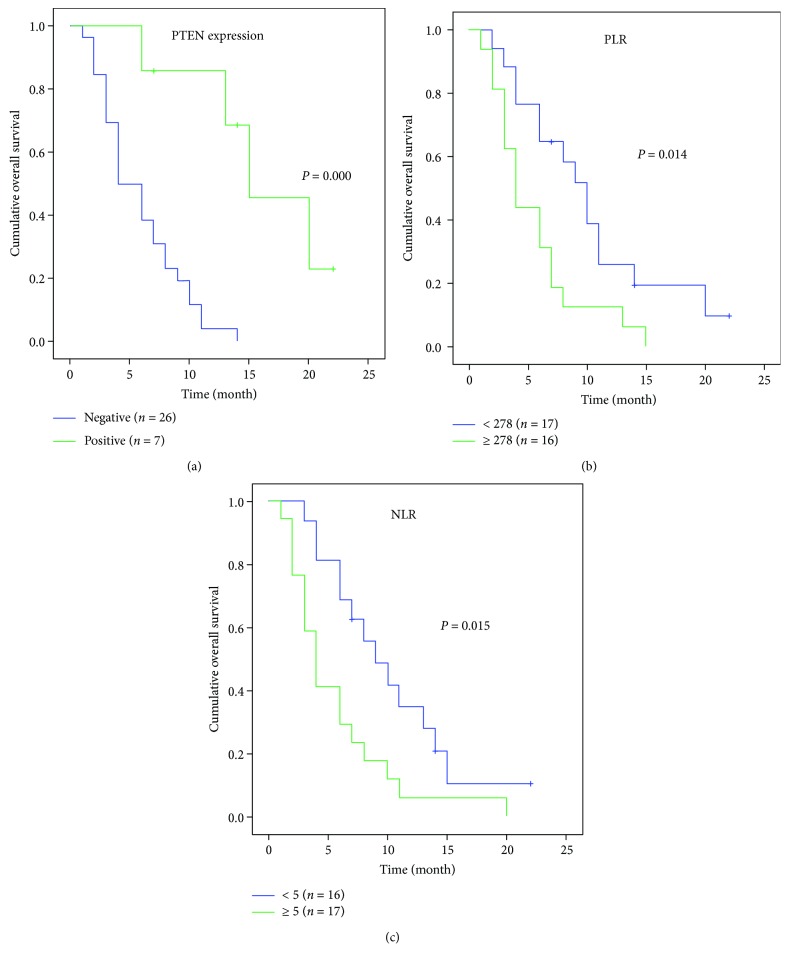
Univariate analyses of overall survival according to PTEN, PLR, and NLR at the time of diagnosis.

Results for the univariate analyses of prespecified individual baseline variables are listed in Table 4. And we set *P* < 0.02 to be statistically significant. PTEN, PLR, and NLR were univariable prognostic predictors (Table 4, Figure 2). Neither asbestos contact nor EGFR, clinical stage, or high platelet count was a significant prognostic predictor in univariable analyses.

The three statistically significant variables in univariate analyses were entered into the multivariate Cox model. Multivariable analysis demonstrated that PTEN expression (HR = 0.070, *P* = 0.001) and PLR (HR = 3.379, *P* = 0.007), but not NLR, were significant independent prognostic predictors (Table 5).

## 4. Discussion

The cause of MPeM is unknown, although evidence implicates asbestos exposure (80%) [22]. We previously found that the incidence of asbestos exposure of patients with MPeM in Eastern China is 93.2% [23]. In the present study, the incidence of asbestos exposure was 87.88%, which is consistent with our previous study. We attribute these findings to the chronic inflammatory reaction to asbestos that eventually leads to cancer.

There has been considerable interest in the association of systemic inflammatory markers and prognosis in both early and advanced carcinoma. Increasing evidence shows that systemic inflammation and immunity play important roles in the occurrence, development, and metastasis of tumors [24]. Persistent inflammation establishes a microenvironment that favors the progression of cancer, and the malignant tumor promotes the inflammatory response. Persistent inflammatory stimulation leads to elevated or reduced numbers of platelets or lymphocytes, respectively, and 20%–60% of patients with cancer suffer from thrombocytosis [25]. Further, we reported that 80.4% of patients with peritoneal mesothelioma patients had elevated numbers of platelets [26].

In the present study, 72.7% of patients had elevated platelet counts, which was consistent with the previous study [27]. The PLR is an important index that reflects a common clinical inflammatory reaction, which can identify patients with tumor-related inflammation. Moreover, in previously performed studies, the PLR was an independent prognostic factor for numerous solid tumors [28–31]. Tagawa et al. reported that in a training cohort with MPuM, multivariate analysis identified sex (*P* = 0.005) and PLR (*P* = 0.049) as independent predictors of overall survival, and patients with a high PLR had a poor prognosis [32]. Tural Onur et al. [7] revealed that the PLR level was a significant prognostic indicator of malignant mesothelioma at diagnosis on complete blood count parameters. In our study, the median PLR value was 278, and there was a significant difference between the survivals of high and low PLR groups (10 months vs 4 months, respectively); meanwhile, PLR was an independent prognostic factor for MPeM. The difference may be explained by the release of the platelet-derived growth factor, vascular endothelial growth factor, and thrombospondin 1. Further, platelets are recruited to the damaged site by diverse cytokines, thereby promoting tumor cell proliferation and adhesion [33].

The NLR can be easily calculated from differential WBC counts obtained through routine procedures, and it was a potential biomarker for stratification in clinical trials and for use in clinical practice. A prognostic role for NLR in MPuM was reported in a number of retrospective series [34]. The cutoff value for NLR used in our analyses was prespecified according to previous reports, an important prerequisite for reliable validation. Our study validated independent prognostic factors, but did not find NLR to be an independent prognostic factor in this cohort of epithelioid MPeM patients. However, in univariate analysis, NLR was a meaningful positive indicator, suggesting that NLR has an impact on the prognosis of epithelioid MPeM.

The imbalance of oncogenes and tumor suppressors, abnormal cell signaling pathways, and abnormal cell cycle regulation plays important roles in the cause and progression of tumors. EGFR promotes cell proliferation and angiogenesis and inhibits apoptosis as well as the escape of tumor cells from the immune response [10]. These cell membrane receptors play a central role in cell proliferation, differentiation, migration, adhesion, and survival [35]. EGFR is an upstream component of the PI3K/mTOR signaling pathway in human neoplasms and is overexpressed in numerous malignant carcinomas (such as breast cancer, gastrointestinal adenocarcinoma, colorectal cancer, and lung cancer [36–39]) and indicates poor prognosis of cancer [40]. Govindan et al. [41] examined paraffin-embedded sections of 24 cases of MM (21 pleural MM) by immunohistochemistry and found EGFR expression in 14 (58%). Edwards et al. [42] found that EGFR expression was a common feature in MM and associated with a favorable prognosis, but it was not an independent prognostic factor when tested against other clinicopathological prognostic factors. In our study, we set *P* < 0.02 for statistical significance in the univariate analysis. We found that EGFR was not statistically significant in the univariate analysis and was inconsistent with the above literature, which of course had a relationship with the *P* value we set. However, studies of a large number of patients with MPeM are therefore required to identify the mechanisms underlying these associations [43].

PTEN, which is mutated at the highest rate in human tumors after P53, resides in human chromosome 10q23.3 and encodes proteins regulating various signal transduction pathways and modulating cell growth processes, cell migration, and apoptosis [44]. PTEN arrests the cell cycle in the G1 phase [45], and the absence of PTEN may activate the PI3K/mTOR pathway. Accordingly, upregulation of the PI3K/mTOR pathway, often through loss of PTEN function, occurs in diverse cancers, including gastrointestinal adenocarcinoma [46], colorectal cancer, and esophageal squamous cell carcinoma [47]. One study demonstrated the prognostic significance of PTEN expression, which was undetectable in 62% of patients [14]. We reported here that PTEN was not detected in 78.79% of the peritoneal tissue samples, which was significantly higher than that reported above. The discrepancy may be explained by differences in the stages of disease and patients' characteristics. Moreover, loss of PTEN expression strongly correlates with shorter survival, and PTEN expression is an independent prognostic biomarker for patients with MPuM [21].

Our findings are in keeping with the extensive body of prior literature of clinical prognostic factors in this disease. For example, survival analysis revealed that patients who expressed PTEN survived significantly longer compared with those with undetectable PTEN expression. Further, consistent with our finding that PTEN expression was an independent prognostic factor for epithelioid MPeM, the regression coefficient was negative, and lower expression was associated with worse prognosis. Therefore, targeting the PI3K/mTOR pathway using inhibitors of components downstream of PTEN may improve patients' outcomes.

Clinical TNM stage can better reflect the degree of tumor development. It has a certain reference value for treatment and prognosis [48]. According to the presence or absence of lymph node and distant metastasis, MPeM is divided into stages I-IV. Although TNM staging is not statistically significant in the univariate analysis, which has something to do with the *P* value we set, the results of this study demonstrate that the prognosis of patients with stage I-II MPeM is significantly better than that of patients with stage III-IV MPeM, suggesting that the higher the stage of TNM, the greater the severity of the disease and the worse the prognosis. Therefore, early diagnosis and treatment are essential for improving survival.

MPeM lacks effective treatments. Intraperitoneal chemotherapy has a certain effect on this disease, and pemetrexed combined with cisplatin has been approved as a first-line therapy for MPeM. Chemotherapy drugs can directly contact the tumor tissue, the local concentration is high, adverse reactions are less than in intravenous chemotherapy, and treatment is safe and effective, as the first choice for nonoperative treatment [49]. In our study, the median survival time was approximately 8 months for the chemotherapy group and 4 months for the nontreatment group. Although chemotherapy was not statistically significant in the univariate analysis, we discovered that systemic chemotherapy obviously prolongs the survival period. Therefore, active treatment is beneficial to the prognosis of patients, and patients should be guided and advocated for chemotherapy in order to prolong the survival time.

## 5. Conclusion

This is the first study to our knowledge to investigate the changes in expression of the biomarkers EGFR, PTEN, PLR, and NLR with the survival of patients with epithelioid MPeM. Our findings indicate that PTEN and PLR are independent prognostic factors of survival. The development of targeted therapies with demonstrable in vitro antitumor activity in epithelioid MPeM will provide a potential new approach for the management of patients afflicted by a tumor. Without a doubt, our research is based on retrospective analysis, so a large number of prospective studies are needed to prove the reliability of the parameters.

## Figures and Tables

**Table 1:** Distribution of descriptive characteristics.

Characteristics	
Age (years)	
Min-max	42-75
Mean ± SD	61.15 ± 8.54
Sex, *n* (%)	
Male	9 (27.27)
Female	24 (72.73)
Asbestos, *n* (%)	
Yes	29 (87.88)
No	4 (12.12)
Platelet, *n* (%)	
High	24 (72.73)
Normal	9 (27.27)
Ascites, *n* (%)	
Yes	27 (81.82)
No	6 (18.18)
Stage, *n* (%)	
I + II	21 (63.64)
III + IV	12 (36.36)
Chemotherapy, *n* (%)	
Yes	14 (42.42)
No	19 (57.58)
PLR	
Min-max	57-1142
Median	278
NLR	
Min-max	1.42-19.00
Median	5.97

**Table 2:** Correlation between EGFR and PTEN expression (cases).

EGFR	PTEN		*r*	*P*
	+	—		
+	1	21	-0.577	0.000
—	6	5		

**Table 3:** Correlation between serum PLR and NLR (cases).

PLR	NLR		*r*	*P*
	<5	≥5		
<278	12	5	0.456	0.008
≥278	4	12		

**Table 4:** Univariate analyses of association of prognostic factors with overall survival of MPeM.

	*N*	No. of deaths	Median survival months (95% CI)	*P* value
Age				
<60	8	6	8 (3.32-12.68)	0.419
≥60	25	24	6 (2.76-9.24)	
Sex				
Female	24	22	6 (3.61-8.39)	0.856
Male	9	8	9 (0-23.61)	
PTEN				
Negative Positive	26	26	4 (2.13-5.87)	0.000
	7	4	15 (8.27-21.73)	
EGFR				
Negative Positive	11	9	11 (8.05-13.95)	0.045
	22	21	4 (2.28-5.72)	
Asbestos				
Absent	4	3	3 (0.00-9.86)	0.869
Present	29	27	6 (3.36-8.64)	
PLT (10^9^/L)				
<300	9	7	10 (7.38-12.62)	0.214
≥300	24	23	4 (2.20-5.80)	
PLR				
<278	17	14	10 (7.54-12.46)	0.014
≥278	16	16	4 (2.70-5.30)	
NLR				
<5	16	13	9 (5.39-12.61)	0.015
≥5	17	17	4 (2.67-5.33)	
LYM (10^3^/*μ*L)				
<0.8	28	26	6 (2.90-9.10)	0.285
≥0.8	5	4	8 (3.71-12.29)	
Ascites				
Absent	6	6	10 (6.61-13.40)	0.406
Present	27	24	6 (3.50-8.50)	
Clinical stage				
I + II	21	18	7 (2.67-11.33)	0.036
III + IV	12	12	4 (0.61-7.40)	
Chemotherapy				
Yes	14	11	8 (4.73-11.27)	0.044
No	19	19	4 (1.44-6.56)	

**Table 5:** COX analyses of prognostic factors with overall survival of MPeM.

		B	SE	Wald	df	Sig	Exp(B)	95.0% CI	
								Lower	Upper
Step 2	PTEN	−2.657	0.811	10.730	1	0.001	0.070	0.014	0.344
	PLR	1.218	0.451	7.282	1	0.007	3.379	1.395	8.181

## Data Availability

The data used to support the findings of this study are available from the corresponding author upon request.
